# Dental health in patients with and without HPV-positive oropharyngeal and tongue cancer

**DOI:** 10.1371/journal.pone.0274813

**Published:** 2022-09-22

**Authors:** Lauri Jouhi, Jenna Sikiö, Anni Suomalainen, Rayan Mroueh, Antti Mäkitie, Jukka H. Meurman

**Affiliations:** 1 Department of Otolaryngology, Head and Neck Surgery, University of Helsinki and Helsinki University Hospital, Helsinki, Finland; 2 Department of Oral and Maxillofacial Diseases, University of Helsinki and Helsinki University Hospital, Helsinki, Finland; 3 Medical Imaging Center, Oral Radiology, University of Helsinki and Helsinki University Hospital, Helsinki, Finland; 4 Research Program in Systems Oncology, University of Helsinki, Helsinki, Finland; 5 Department of Clinical Sciences, Division of Ear, Nose and Throat Diseases, Intervention and Technology, Karolinska Institutet and Karolinska Hospital, Stockholm, Sweden; University of Catania, ITALY

## Abstract

**Background:**

Human papilloma virus is associated with oral and oropharyngeal cancer. Our aim was to examine oral health in patients with oropharyngeal (OPSCC) and oral tongue cancer (OTSCC), expecting better oral health among OPSCC patients.

**Material and methods:**

Fifty-five OPSCC patients with known HPV status and 59 OTSCC patients were randomly selected from a list of consecutive patients of the Helsinki University Hospital, Finland. Oral health was assessed from panoramic jaw radiographs. Total Dental Index (TDI) summarizing the dental health status was calculated and Finnish population study data were used for comparison. Descriptive statistics were used for analyses.

**Results:**

Patients with HPV-positive OPSCC had higher periapical lesion index compared with HPV-negative OPSCC patients or with OTSCC patients. Residual roots were more common among OPSCC patients compared with OTSCC patients, because of their higher occurrence among HPV-negative OPSCC patients compared with OTSCC patients. Similarly, modified TDI score was significantly higher among OPSCC patients than among OTSCC patients, because of higher TDI score among HPV-negative OPSCC patients compared with OTSCC patients. OPSCC patients more often used a removable prosthesis than OTSCC patients. Dental health of the cancer patients was poorer when compared with the population data.

**Conclusions:**

Our study hypothesis was only partly confirmed. Periapical lesions were more prevalent among HPV-positive OPSCC patients, compared with the other groups. The number of residual roots was higher among HPV-negative subgroup. Thus, OPSCC patients had worse oral health parameters than OTSCC patients.

## Introduction

Head and neck cancers (HNCs) represent a global public health problem with an estimated 355 000 new cases of and oral cancer and 93 000 oral pharyngeal cancer, respectively, occurring in 2018 worldwide [1]. Smoking and alcohol consumption are the most important risk factors for these tumors, and they typically present at older age. However, an increasing subset of especially oropharyngeal cancers (OPSCC) is related to human papillomavirus (HPV) infection [2]. The average age of patients with HPV-related cancer seems to be lower than that of the non-HPV-related group and these populations also differ in their drinking and smoking habits [3]. In a recent study on OPSCC patients, smoking and heavy alcohol consumption were significantly more common among HPV−/p16− OPSCC patients but also rather common among HPV+/p16+ patients [4]. Furthermore, as another recent study showed, there seems to be a shift towards the HPV-related oral and oropharyngeal cancer when compared with HPV-negative cases, in other words, the incidence of HPV+ cases are increasing [5]. Predisposing factors also include nutritional deficits, occupational hazards, anemia, fungal and certain other viral infections, hereditary factors, immunosuppression, and potentially malignant disorders such as leukoplakia and oral lichen planus [6, 7].

There is cumulative evidence that oral infections and poor oral hygiene are associated with HNCs but the exact mechanism remains unclear [8–16]. Most clearly poor oral hygiene manifests itself in the oral cavity as caries and periodontitis. Periodontitis is a chronic infection and causes chronic inflammation locally in the oral cavity and also has adverse effects on systemic health. An association has been suggested between periodontitis and certain subsites of HNC, and it seems to be strongest for oral cavity, followed by oropharynx and larynx [11, 12]. Substantial evidence supports the role of chronic infection and inflammation in initiating and promoting carcinogenesis in general [17]. Furthermore, it has even been proposed that cariogenic bacterial flora might be a protective factor against cancer [8]. Evidence also suggests that carcinogenic effect of alcohol is mediated by its first metabolite–acetaldehyde. Oral bacteria can enzymatically convert ethanol to acetaldehyde, and this has been proposed as a mechanism between poor dental status and increased oral cancer risk [9, 18]. Further, poor oral hygiene-associated bacterial overgrowth and ecological shift towards acetaldehyde producing bacteria may contribute to carcinogenesis. There is also a correlation between poor oral hygiene and the number of extracted teeth with the prevalence of HPV, which finding emphasizes the importance for maintaining good oral health also in this regard [19].

The primary aim of this study was to examine dental health in a subset of HNC patients, namely those with oral tongue cancer (OTSCC) and those with OPSCC whose HPV status had been analyzed. Our secondary aim was to compare these two tumor sites in terms of oral health as our hypothesis was that the OPSCC patients would have better oral health status. Radiologic assessment, *i*.*e*. panoramic tomography taken at the time of cancer diagnosis, was used to evaluate the accumulated burden of dental diseases. A Finnish population study data on dental health were used for comparison.

## Subjects and methods

### Patients

All consecutive patients diagnosed with OPSCC (n = 132) or OTSCC (n = 90) between the years 2005 and 2008 at the Helsinki University Hospital, Helsinki, Finland were identified from hospital registries. For further assessment, 138 patients (69 patients with OPSCC and 69 patients with OTSCC) were randomly selected from the consecutive patient groups. Oral health at diagnosis among the study population was assessed by reviewing panoramic radiographs. Patients with no panoramic radiograph at the time of diagnosis or patients who had previously received radiotherapy to the head and neck region (14 OPSCC and 10 OTSCC patients) were considered ineligible for our analysis. Eventually, the study sample consisted of 114 patients (55 OPSCC patients and 59 OTSCC patients).

### Panoramic radiographs

All panoramic radiographs were analyzed by two persons (J.S. and an experienced dental radiologist A.S.). The other reader (J.S.) analysed the images first independently, followed by consensus reading. For practical reasons the examiners were not blinded to cancer status. The following variables were determined: number of teeth in the upper and lower jaw, carious lesions extending to dentin, root fragments, periapical lesions (apical periodontitis or widening of the periodontal space), endodontically treated teeth, technically inadequate endodontic treatments (the gap between filling and apex more than 3 mm, pulpotomy, or overfilling), and furcation lesions, as well as horizontal bone loss, vertical bone pockets, pericoronitis (radiolucent follicle around third molars with a diameter of 3 mm or more), and dental calculus. The Total Dental Index introduced by Mattila et al. [20] was modified and used to illustrate the severity of dental disease burden.

### Clinicopathological variables

Details on covariates including age, gender, smoking, alcohol consumption, denture wear, record of clinical mucosal lesions in oral cavity apart from the tumor, existing diagnoses and medications was manually collected from medical records. TNM stage and tumor grade were recorded for all patients and tumor HPV status for patients with OPSCC only. Existing diagnoses were categorized according to the 10th revision of the World Health Organization International Classification of Diseases (ICD-10) and information on medications according to a specific national drug classification (www.terveysportti.fi). Ex-smokers with smoking cessation of over five years were considered non-smokers. All patients in both groups had histopathological squamous cell carcinoma either in the oral cavity or oropharynx. The management of all patients was discussed at a multidisciplinary tumor board at the university hospital and was based on a national treatment protocol. It typically consisted of surgery, (chemo)radiotherapy or their combination.

### Statistical data analysis

We used SPSS Version 25.0 (SPSS, Inc., Chicago, IL, USA) for statistical data analysis. The cross-tabulation of categorical variables was performed using χ^2^ test with asymptotic or exact p-value when best appropriate. Independent samples T-test was used to compare means of normally distributed continuous variables between two independent groups. Mann-Whitney U test was used to compare two independent groups with skewed distribution. A two-sided *P* value <0.05 was considered statistically significant.

### Ethics statement

This study is a retrospective patient chart review and according to the Finnish Law no Research Ethics Board approval was needed. An institutional research permission was granted (§121, 02.07.2014). All procedures performed in this study involving information on patients were in accordance with the ethical standards of the institutional and/or national research committee and with the 1964 World Medical Association Declaration of Helsinki and its later amendments or comparable ethical standards.

### Informed consent

As this study was a retrospective chart review, no informed consent was needed according to the Finnish legislation.

## Results

### Clinicopathological characters of the OPSCC and OTSCC patients

Table 1 shows the baseline characteristics of the study population. The mean age of the whole study population was 59.5 years (range 30.4–89.6 years), and the male-to-female ratio was approximately 2:1. Of the patients for whom the information was available 58% were smokers and 37% were heavy alcohol users. Stage IV and grade 3 tumors were most frequently seen. The median number of drugs in daily use was 1 (range 0–9). Of the patients, 11% were edentulous, 23% had a record of removable prosthesis and 10% had mucosal lesions (other than cancer) in the oral cavity. Ten percent of them had a former cancer diagnosis (*i*.*e*. C00-D48) and 8% had been previously diagnosed with a head and neck area digestive system diagnosis (*i*.*e*. K00-K14).

**Table 1:** Baseline clinic-pathological characteristics of the patients with oropharyngeal (OPSCC) or oral tongue (OTSCC) squamous cell carcinoma.

	Total	OPSCC	OTSCC	*P*
No of patients (%)	114 (100)	55 (48.2)	59 (51.8)	
Age yrs. (range)	59.5 (30.4–89.6)	59.5 (33.3–89.6)	59.5 (30.4–85.7)	0.980
Gender				0.174
Male	78 (68.4)	41 (74.5)	37 (62.7)	
Female	36 (31.6)	14 (25.5)	23 (37.3)	
Edentulous	12 (10.5)	5 (9.1)	7 (11.9)	0.630
Smoking				0.285
Yes	56 (58.3)	30 (63.8)	26 (53.1)	
No	40 (41.7)	17 (36.2)	23 (46.9)	
Missing	18	8	10	
Heavy alcohol consumption				0.432
Yes	29 (36.7)	13 (32.5)	16 (41.0)	
No	50 (63.3)	27 (67.5)	23 (59.0)	
Missing	35	15	20	
TNM stage				<0.001
I	21 (18.8)	4 (7.4)	17 (29.3)	
II	32 (28.6)	7 (13.0)	25 (43.1)	
III	11 (9.8)	6 (11.1)	5 (8.6)	
IV	48 (42.9)	37 (68.5)	11 (19.0)	
Missing	2	1	1	
Grade				0.002
1	23 (26.7)	8 (14.8)	15 (46.9)	
2	30 (34.9)	19 (35.2)	11 (34.4)	
3	33 (38.4)	27 (50.0)	6 (18.8)	
Missing	28	1	27	
HPV status				
Positive		21 (46.7)		
Negative		24 (53.3)		
Missing		10		
Median number of drugs in use (range)	1 (0–9)	1 (0–9)	2 (0–9)	0.411
Mucosal lesion	11 (9.6)	2 (3.6)	9 (15.3)	0.036
C00-D48	11 (9.6)	2 (3.6)	9 (15.3)	0.036
K00-K14	9 (7.9)	1 (1.8)	8 (13.6)	0.033

The OPSCC and OTSCC groups did not have a statistically significant difference with regard to age, gender, edentulousness, smoking, heavy alcohol consumption, or number of drugs in use. However, TNM stage and tumor grade were significantly higher in the OPSCC group. Removable prostheses were significantly more frequent in the OPSCC group and mucosal lesions in oral cavity were significantly more frequent in the OTSCC group. There were more C00-D48 category diagnoses and K00-K14 category diagnoses in the OTSCC group. Twenty-one OPSCC patients had a HPV-positive tumor while 24 had a HPV-negative tumor (Table 2).

**Table 2:** Dental indexes of the study population with oropharyngeal (OPSCC) and oral tongue (OTSCC) squamous cell carcinoma.

	Total	OPSCC			OTSCC	*P*
		Total	HPV+	HPV-		
No of patients (%)	114 (100)	55 (48.2)	21 (46.7)	24 (53.3)	59 (51.8)	
Caries						0.129^a^
0	36 (31.6)	12 (21.8)	7 (33.3)	4 (16.7)	24 (40.7)	0.079^b^
1	40 (35.1)	22 (40.0)	9 (42.9)	11 (45.8)	18 (30.5)	0.703^c^
2	22 (19.3)	13 (23.6)	5 (23.8)	4 (16.7)	9 (15.3)	0.136^d^
3	16 (14.0)	8 (14.5)	0 (0.0)	5 (20.8)	8 (13.6)	
Periodontitis						0.608^a^
0	35 (30.7)	16 (29.1)	6 (28.6)	7 (29.2)	19 (32.2)	0.738^b^
1	37 (32.5)	17(30.9)	8 (38.1)	8 (33.3)	20 (33.9)	1.000^c^
2	36 (31.6)	19 (34.5)	7 (33.3)	7 (29.2)	17 (28.8)	0.695^d^
3	6 (5.3)	3 (5.5)	0 (0.0)	2 (8.3)	3 (5.1)	
Periapical lesions						0.123^a^
0	36 (31.6)	13 (23.6)	2 (9.5)	8 (33.3)	23 (39.0)	**0.029** ^b^
1	63 (55.3)	33 (60.0)	14 (66.7)	14 (58.3)	30 (50.8)	**0.013** ^**c**^
2	8 (7.0)	5 (9.1)	2 (9.5)	2 (8.3)	3 (5.1)	1.000^d^
3	7 (6.1)	4 (7.3)	3 (14.3)	0 (0.0)	3 (5.1)	
Pericoronitis						0.482^a^
0	113 (99.1)	54 (98.2)	21 (100.0)	23 (95.8)	59 (100.0)	1.000^b^
1	1 (0.9)	1 (1.8)	0 (0.0)	1 (4.2)	0 (0.0)	0.289^d^
Residual roots						**0.006** ^a^
0	98 (86.0)	42 (76.4)	17 (81.0)	19 (79.2)	56 (94.9)	0.720^b^
1	7 (6.1)	5 (9.1)	2 (9.5)	2 (8.3)	2 (3.4)	0.107^c^
2	5 (4.4)	5 (9.1)	2 (9.5)	1 (4.2)	0 (0.0)	**0.032** ^d^
≥3	4 (3.5)	3 (5.5)	0 (0.0)	2 (8.3)	1 (1.7)	
Furcation lesions						0.412^a^
0	81 (71.1)	35 (63.6)	12 (57.1)	17 (70.8)	46 (78.0)	0.388^b^
1	14 (12.3)	10 (18.2)	4 (19.0)	4 (16.7)	4 (6.8)	0.281^c^
2	6 (5.3)	4 (7.3)	2 (9.5)	1 (4.2)	2 (3.4)	1.000^d^
≥3	13 (11.4)	6 (10.9)	3 (14.3)	2 (8.3)	7 (11.9)	
Vertical bone pockets						0.916^a^
						0.506^b^
0	53 (46.5)	21 (38.2)	7 (33.3)	10 (41.7)	32 (54.2)	0.692^c^
1	24 (21.1)	17 (30.9)	6 (28.6)	8 (33.3)	7 (11.9)	0.849^d^
2	10 (8.8)	7 (12.7)	4 (19.0)	2 (8.3)	3 (5.1)	
≥3	27 (23.7)	10 (18.2)	4 (19.0)	4 (16.7)	17 (28.8)	
Endodontic treatments						0.260^a^
						0.310^b^
0	45 (39.5)	20 (36.4)	4 (19.0)	9 (37.5)	25 (42.4)	0.054^c^
1–2	33 (28.9)	15 (27.3)	8 (38.1)	6 (25.0)	18 (30.5)	0.534^d^
3–4	25 (21.9)	13 (23.6)	5 (23.8)	7 (29.2)	12 (20.3)	
≥5	11 (9.6)	7 (12.7)	4 (19.0)	2 (8.3)	4 (6.8)	
Removable prostheses						**0.047** ^a^
						0.142^b^
No	88 (77.2)	38 (69.1)	18 (85.7)	16 (66.7)	50 (84.7)	1.000^c^
Yes	26 (22.8)	17 (30.9)	3 (14.3)	8 (33.3)	9 (15.3)	0.078^d^
TDI score						**0.018** ^a^
0–2	40 (35.1)	12 (21.8)	8 (38.1)	4 (16.7)	28 (47.5)	0.173^b^
3–5	65 (57.0)	38 (69.1)	12 (57.1)	18 (75.0)	27 (45.8)	0.678^c^
6–8	9 (7.9)	5 (9.1)	1 (4.8)	2 (8.3)	4 (6.8)	**0.029** ^d^

^a^ OPSCC vs. OTSCC

^b^ HPV+ OPSCC vs. HPV- OPSCC

^c^ HPV+ OPSCC vs. OTSCC

^d^ HPV- OPSCC vs. OTSCC

### Dental status among OPSCC and OTSCC patients

On average, patients had 19 teeth, of which 10 were in mandible and nine in maxilla. Half of them i.e. 49% had carious lesions, 14% had residual roots, 54% had vertical bone pockets, 29% had furcation lesions, and 40% had periapical lesions. Of all patients 61% had at least one endodontically treated tooth, and 65% out of these had at least one technically inadequate endodontic treatment. Horizontal bone loss was recorded in 69%, and of those 84% had more than one affected tooth. Comparing the dental status parameters of our patients with known results from Finnish population register studies, the prevalence of caries was higher among the cancer patients; in the mean 49% of patients had at least one carious lesion compared with 32% of the general population, while no difference was found in periodontitis parameters in this respect [21, 22].

The HNC subgroups appeared to be fairly similar in terms of their dental variables. Interestingly, patients with HPV-positive OPSCC had higher periapical lesion index compared with HPV-negative OPSCC patients and with OTSCC patients. The number of residual roots was also higher among OPSCC patients than among OTSCC patients. The subgroup analysis revealed that the number of residual roots was higher among HPV-negative OPSCC patients compared with those with OTSCC. Similarly, modified TDI score was significantly higher among OPSCC patients than among OTSCC, because of higher number of residual roots among HPV-negative OPSCC than among OTSCC. In addition, use of removable prostheses was more frequent among OPSCC patients than among OTSCC patients.

## Discussion

We retrospectively reviewed a series of 114 panoramic tomography studies of OPSCC and OTSCC patients to evaluate their oral health status at the time of cancer diagnosis. In most aspects, the two groups were not found to be substantially different from each other. Periodontal disease–whether assessed by clinical attachment loss, alveolar bone loss, or probing pocket depth–seems to associate with elevated risk for HNC [11, 15, 23]. On the other hand, dental caries may act inversely, protecting from cancer [24]. Periodontal diseases and dental caries are common bacterial diseases in general population associated with dental plaque and poor oral hygiene [21, 22, 25–28]. But they are counterparts to each other in the sense that cariogenic bacterial flora is associated with periodontal health [29, 30]. Missing teeth are often used as a substitute for periodontitis, but in a retrospective study design it is impossible to know the reason for tooth loss. In the absence of control patients, we were not able to establish whether or not our findings regarding caries and periodontal disease support findings from previous studies. Nevertheless, caries and periodontal disease indicate poor oral hygiene.

Our hypothesis was that the OPSCC patients would have better oral health status. Contrary to that, considerable similarity of dental variables between OPSCC and OTSCC groups was found. However, this finding might be explained by the close anatomical relation of OTSCC and OPSCC and their common risk factors. If elevated acetaldehyde levels in saliva mediate the carcinogenic effect of poor dental health, as predicted by Homann et al. [8, 18], it would be rather straightforward to assume that while saliva is swallowed the effect will be spread from oral cavity also to oropharynx. Periodontal disease, on the other hand, is known to influence systemic health and to cause disease beyond the oral cavity [31]. According to our analysis, the differences between OPSCC and OTSCC in dental variables were scarce. However, findings of higher number of periapical lesions and residual roots among OPSCC patients may indicate slightly better dental health among OTSCC patients, but the reason for this remains unclear.

The main difference in risk factors between OPSCC and OTSCC seems to be the proportion of HPV induced carcinomas. Findings by Bui et al. [32] suggest that poor oral health may increase the odds of oral HPV infection. Thus, poor oral health would indirectly increase the risk of oropharyngeal cancers [33]. A review article by Combes and Franceschi [34] suggested that the probability of a HPV-attributable fraction of cancer in the oral cavity was estimated to be at least 5-fold lower compared to that in the oropharynx, and HPV prevalence among oral cavity cancer to be about 3%. Worldwide the trend seems to be that even though the use of tobacco declines the incidence of OPSCC rises. This is suggested to be attributable to increased oral HPV infections [35, 36]. Patients with HPV-positive tumors are usually younger and less likely to have a history of smoking or alcohol use than patients with HPV-negative tumors [4]. HPV-positive tumors are diagnosed at more advanced stages, but prognosis among HPV-positive subgroup tends to be better than that of HPV-negative subgroup [36, 37].

We screened the medical records for former head and neck area (K00-K14) and oral cavity mucosal lesions occurring concurrently with the actual tumor, as they are mucosal conditions with a risk of malignant transformation [7, 38]. The fact that OPSCC group had less former K00-K14 category diagnoses and also less oral mucosal lesions recorded at the time of cancer diagnosis may be related to absence of field cancerization effect in HPV-related OPSCC [39]. In the literature the association between mucosal lesions and cancer is mainly studied in oral cavity [7, 38], although it is known that some of these potentially malignant disorders can occur even in oesophagus [40]. However, there is also an assumed causality between these disorders and oropharyngeal cancers [41]. In addition, former cancer and tumor diagnoses (C00-D48) were more common among OTSCC patients. The finding that OTSCC patients had more oral cavity mucosal lesions, K00-K14 and C00-D48 diagnoses, may be related to higher exposure to tobacco carcinogens, heavy drinking lifestyle, and higher occurrence of comorbidities among the OTSCC patients, while OPSCC patients are increasingly influenced by HPV.

The present study included patients from consecutive series of OPSCC and OTSCC patients during the time period when the incidence of HPV-related OPSCC was rising in Finland. All patients received diagnostic evaluation and treatment at a public tertiary care center, and the impact of socio-economic factors in the management of these patients remains minor which is a strength. However, the retrospective setting of this study partially complicated recoding of the data, as some information remained inexact or unmentioned in the medical records. Thus, the sample sizes used in the statistical analyses were limited, which is a weakness in our study. Finally, it should be mentioned that HPV vaccinations are expected to reduce the number of OPSCC cases in the future [42]. However, beyond those young generations now being vaccinated there remain many age groups with risk for developing HPV-related cancer. Focus should thus be placed on careful clinical oral and oropharyngeal examination to identify any suspicious lesions.

## Conclusion

In conclusion, the differences in dental health between the OPSCC and OTSCC patients remained relatively limited with some factors indicating better dental health among OTSCC patients. These findings call for further validation in larger cohorts with a case-control or longitudinal setting.

## References

[1] Bray F, Ferlay J, Soerjomataram I, Siegel RL, Torre LA, Jemal A. Global cancer statistics 2018: GLOBOCAN estimates of incidence and mortality worldwide for 36 cancers in 185 countries. CA Cancer J Clin. 2018; 68: 394–424. doi: 10.3322/caac.21492 30207593

[2] Marur S D’Souza G, Westra WH, et al. HPV-associated head and neck cancer: A virus-related cancer epidemic. Lancet Oncol. 2010; 11: 781–9. doi: 10.1016/S1470-2045(10)70017-6 20451455 PMC5242182

[3] Chaturvedi AK. Epidemiology and clinical aspects of HPV in head and neck cancers. Head Neck Pathol. 2012; 6 (suppl 1): S16–24. doi: 10.1007/s12105-012-0377-0 22782220 PMC3394159

[4] Carpén T, Sjöblom A, Lundberg M, Haglund C, Markkola A, Syrjänen S, et al. Presenting symptoms and clinical findings in HPV-positive and HPV-negative oropharyngeal cancer patients. Acta Otolaryngol. 2018;138: 513–8. doi: 10.1080/00016489.2017.1405279 29161981

[5] Dos Santos Menedes F, Dias de Oliviera Latorre M, De Souza Conceicao GM, Curado MP, Ferreira Antunes JL, Toporcov TN. The emerging risk of oropharyngeal and oral cavity cancer in HPV-related subsites in young people in Brazil. PLoS ONE. 2020;15. doi: 10.1371/journal.pone.0232871 32407339 PMC7224475

[6] Chi AC, Day TA, Neville BW. Oral cavity and oropharyngeal squamous cell carcinoma—an update. CA Cancer J Clin. 2015;65: 401–21. doi: 10.3322/caac.21293 26215712

[7] Goodson ML, Sloan P, Robinson CM, et al. Oral precursor lesions and malignant transformation—who, where, what, and when? Br J Oral Maxillofac Surg. 2015; 53: 831–55. doi: 10.1016/j.bjoms.2015.08.268 26388071

[8] Homann N, Tillonen J, Rintamäki H, Salaspuro M, Lindqvist C, Meurman JH. Poor dental status increases acetaldehyde production from ethanol in saliva: A possible link to increased oral cancer risk among heavy drinkers. Oral Oncol. 2001; 37: 153–8. doi: 10.1016/s1368-8375(00)00076-2 11167142

[9] Rosenquist K, Wennerberg J, Schildt EB, Bladström A, Hansson GB, Andersson G. Oral status, oral infections and some lifestyle factors as risk factors for oral and oropharyngeal squamous cell carcinoma. A population-based case-control study in southern Sweden. Acta Oto-Laryngologica. 2005; 125: 1327–36. doi: 10.1080/00016480510012273 16303683

[10] Rezende CP, Ramos MB, Daguíla CH, Dedivitis RA, Rapoport A. Oral health changes in with oral and oropharyngeal cancer. Braz J Otorhinolaryngol. 2008; 74: 596–600. doi: 10.1016/s1808-8694(15)30609-1 18852988 PMC9442110

[11] Tezal M, Sullivan MA, Hyland A, et al. Chronic periodontitis and the incidence of head and neck squamous cell carcinoma. Cancer Epidemiol Biomarkers Prev. 2009;18: 2406–12. doi: 10.1158/1055-9965.EPI-09-0334 19745222

[12] Shigeishi H, Sugiyama M, Ohta K. Relationship between the prevalence of oral human papillomavirus DNA and periodontal disease (Review). Biomed Rep. 2021:14. doi: 10.3892/br.2021.1416 33728046 PMC7953200

[13] Meurman JH. Infectious and dietary risk factors of oral cancer. Oral Oncol. 2010;6: 411–3. doi: 10.1016/j.oraloncology.2010.03.003 20381409

[14] Chang JS, Lo HI, Wong TY, Huang CC, Lee WT, Tsai ST, et al. Investigating the association between oral hygiene and head and neck cancer. Oral Oncol. 2013; 49: 1010–7. doi: 10.1016/j.oraloncology.2013.07.004 23948049

[15] Moraes RC, Dias FL, Figueredo CM, Fischer RG. Association between chronic periodontitis and oral/oropharyngeal cancer. Braz Dent J. 2016; 27: 261–6. doi: 10.1590/0103-6440201600754 27224557

[16] Hashim D, Sartori S, Brennan P, Curado MP, Wünsch-Filho V, Divaris K, et al. The role of oral hygiene in head and neck cancer: results from International Head and Neck Cancer Epidemiology (INHANCE) consortium. Ann Oncol. 2016;27: 1619–25. doi: 10.1093/annonc/mdw224 27234641 PMC4959929

[17] Coussens LM, Werb Z. Inflammation and cancer. Nature. 2002; 420: 860–7. doi: 10.1038/nature01322 12490959 PMC2803035

[18] Homann N, Tillonen J, Meurman JH, Rintamäki H, Lindqvist C, Rautio M, et al. Increased salivary acetaldehyde levels in heavy drinkers and smokers: a microbiological approach to oral cavity cancer. Carcinogenesis. 2000; 21: 663–8. doi: 10.1093/carcin/21.4.663 10753201

[19] Dalla Torre D, Burtscher D, Sölder E, Rasse M, Puelacher W. The correlation between the quality of oral hygiene and oral HPV infection in adults: a prospective cross-sectional study. Clin Oral Investig. 2019;23:179–185. doi: 10.1007/s00784-018-2425-y 29574499

[20] Mattila KJ, Nieminen MS, Valtonen VV, Rasi VP, Kesäniemi YA, Syrjälä SL, et al. Association between dental health and acute myocardial infarction. Brit Med J. 1989; 298: 779–81. doi: 10.1136/bmj.298.6676.779 2496855 PMC1836063

[21] Aromaa A. Terveys ja toimintakyky Suomessa: Terveys 2000 -tutkimuksen perustulokset. Available at: http://urn.fi/URN:ISBN:951-740-262-7

[22] Koskinen S, Lundqvist A, Ristiluoma N. Terveys, toimintakyky ja hyvinvointi Suomessa 2011. Available at: http://urn.fi/URN:ISBN:978-952-245-769-1

[23] Tezal M, Grossi SG, Genco RJ. Is periodontitis associated with oral neoplasms? J Periodontol. 2005; 76: 406–10. doi: 10.1902/jop.2005.76.3.406 15857075

[24] Tezal M, Scannapieco FA, Wactawski-Wende J, Meurman JH, Marshall JR, Rojas IG, et al. Dental caries and head and neck cancers. JAMA Otolaryngol Head Neck Surg. 2013; 139: 1054–60. doi: 10.1001/jamaoto.2013.4569 24030728

[25] Selwitz RH, Ismail AI, Pitts NB. Dental caries. Lancet. 2007;369: 51–9. doi: 10.1016/S0140-6736(07)60031-2 17208642

[26] Eke PI, Dye BA, Wei L, Slade GD, Thornton-Evans GO, Borgnakke WS, et al. Update on prevalence of periodontitis in adults in the United States: NHANES 2009 to 2012. J Periodontol. 2015; 86: 611–62. doi: 10.1902/jop.2015.140520 25688694 PMC4460825

[27] Sheiham A, Netuveli GS. Periodontal diseases in Europe. Periodontol 2000. 2002; 29: 104–21. doi: 10.1034/j.1600-0757.2002.290106.x 12102705

[28] Dye BA. Global periodontal disease epidemiology. Periodontol 2000. 2012; 58: 10–25. doi: 10.1111/j.1600-0757.2011.00413.x 22133364

[29] Wade WG. The oral microbiome in health and disease. Pharmacol Res. 2013; 69: 137–43. doi: 10.1016/j.phrs.2012.11.006 23201354

[30] Kõll-Klais P, Mändar R, Leibur E, Marcotte H, Hammarström L, Mikelsaar M. Oral lactobacilli in chronic periodontitis and periodontal health: species composition and antimicrobial activity. Oral Microbiol Immunol. 2005; 20: 354–61. doi: 10.1111/j.1399-302X.2005.00239.x 16238595

[31] Han YW, Houcken W, Loos BG, Schenkein HA, Tezal M. Periodontal disease, atherosclerosis, adverse pregnancy outcomes, and head-and-neck cancer. Adv Dent Res. 2014; 26: 47–55. doi: 10.1177/0022034514528334 24736704 PMC10477771

[32] Bui TC, Markham CM, Ross MW, Mullen PD. Examining the association between oral health and oral HPV infection. Cancer Prev Res. (Phila). 2013; 6: 917–24. doi: 10.1158/1940-6207.CAPR-13-0081 23966202

[33] Kreimer AR, Clifford GM, Boyle P, Franceschi S. Human papillomavirus types in head and neck squamous cell carcinomas worldwide: a systematic review. Cancer Epidemiol Biomarkers Prev. 2005; 14: 467–75. doi: 10.1158/1055-9965.EPI-04-0551 15734974

[34] Combes JD, Franceschi S. Role of human papillomavirus in non-oropharyngeal head and neck cancers. Oral Oncol. 2014; 50: 370–9. doi: 10.1016/j.oraloncology.2013.11.004 24331868

[35] Hammarstedt L, Dahlstrand H, Lindquist D, et al. The incidence of tonsillar cancer in Sweden is increasing. Acta Otolaryngol. 2007; 127: 988–92. doi: 10.1080/00016480601110170 17712680

[36] Chaturvedi AK, Engels EA, Anderson WF, Gillison ML. Incidence trends for human papillomavirus-related and unrelated oral squamous cell carcinomas in the United States. J Clin Oncol. 2008; 26: 612–9. doi: 10.1200/JCO.2007.14.1713 18235120

[37] López RVM, Levi JE, Eluf-Neto J, et al. Human papillomavirus (HPV) 16 and the prognosis of head and neck cancer in a geographical region with a low prevalence of HPV infection. Cancer Causes Control. 2014; 25: 461–71. doi: 10.1007/s10552-014-0348-8 24474236

[38] Aghbari SMH, Abushouk AI, Attia A, Elmaraezy A, Menshawy A, Ahmed MS, et al. Malignant transformation of oral lichen planus and oral lichenoid lesions: A meta-analysis of 20095 patient data. Oral Oncol. 2017; 68: 92–102. doi: 10.1016/j.oraloncology.2017.03.012 28438300

[39] Rietbergen MM, Braakhuis BJ, Moukhtari N, Bloemena E, Brink A, Sie D, et al. No evidence for active human papillomavirus (HPV) in fields surrounding HPV-positive oropharyngeal tumors. J Oral Pathol Med. 2014; 43: 137–42. doi: 10.1111/jop.12123 24118314

[40] Quispel R, van Boxel OS, Schipper ME, Sigurdsson V, Canninga-van Dijk MR, Kerckhoffs A, et al. Schwartz MP. High prevalence of esophageal involvement in lichen planus: a study using magnification chromoendoscopy. Endoscopy. 2009; 41: 187–93. 19280529 10.1055/s-0028-1119590

[41] van der Waal I. Potentially malignant disorders of the oral and oropharyngeal mucosa; terminology, classification and present concepts of management. Oral Oncol. 2009; 45: 317–23. doi: 10.1016/j.oraloncology.2008.05.016 18674954

[42] Lehtinen T, Elfström KM, Mäkitie A, Nygård M, Vänskä S, Pawlita M, et al. Elimination of HPV-associated oropharyngeal cancers in Nordic countries. Prev Med. 2021;144:106445. doi: 10.1016/j.ypmed.2021.106445 33678237

